# Transcriptome analysis of arterial and venous circulating miRNAs during hypertension

**DOI:** 10.1038/s41598-021-82979-7

**Published:** 2021-02-10

**Authors:** Ling Jin, Min Li, Hao Wang, Zhongnan Yin, Li Chen, Yang Zhou, Yongzheng Han, Qinghua Cui, Yuan Zhou, Lixiang Xue

**Affiliations:** 1 Center of Basic Medical Research, Institute of Medical Innovation and Research, Peking University Third Hospital, Beijing, 100191 China; 2 Department of Anesthesiology, Peking University Third Hospital, Beijing, 100191 China; 3 Department of Biomedical Informatics, School of Basic Medical Sciences, Peking University, Beijing, 100191 China

**Keywords:** Cardiovascular biology, Biomarkers

## Abstract

Most current circulating miRNA biomarkers are derived from peripheral venous blood, whereas miRNA deregulation in arterial blood in disease conditions has been largely ignored. To explore whether peripheral venous blood miRNAs could represent a bona fide specific miRNA deregulation pattern, we selected hypertension, a disease that is particularly associated with vessels, as the model. Circulating miRNA profiles of arterial and venous blood from spontaneously hypertensive (SHR) rats and their corresponding controls (i.e., WKY rats) were investigated by next-generation miRNA sequencing. Little miRNAs were observed between arterial and venous circulating miRNAs in WKY rats. Interestingly, this number was enhanced in SHR hypertensive rats. Bioinformatical analysis of disease association, enriched target genes and the regulatory transcription factors of these differentially expressed miRNAs implied a potential functional link with cardiovascular disease-related functions. Comparisons between arterial and venous miRNAs in hypertension-versus-control conditions also revealed prominent disease association of circulating miRNAs and their target genes in arteries but not in veins. Moreover, a young non-hypertensive animal model in SHR background (i.e. JSHR) was used as a second control for SHR. Additional transcriptomic analysis and droplet digital PCR validation of arterial and venous deregulated miRNAs among SHR and its two controls (WKY, JSHR) revealed a noticeable consensus of artery-deregulated miRNAs in hypertension and two novel arterial circulating signatures (miR-455-3p and miR-140-3p) of hypertension. These results suggest the necessity of re-evaluating the efficacy of certain venous miRNAs identified in previous studies as potential biomarkers in cardiovascular diseases or a wider disease spectrum.

## Introduction

Blood-borne miRNAs (aka circulating miRNAs) are a group of circulating RNA molecules that are recognized as promising biomarkers for screening, diagnosis, prediction and biological stratification^1^. These miRNAs are short ncRNAs that are significantly concentrated outside cells, exhibit rapid release dynamics and are extremely stable^2^. Some of them are organ-specific^3^ or cell-type-specific^4^. All these traits demonstrate the deregulated or differentially expressed circulating miRNAs as ideal biomarkers that could reflect pathophysiological changes.

MiRNAs from plasma and serum have been selected for the analyses of circulating miRNAs. However, since the coagulation process may affect miRNA profiles in serum samples, plasma samples were mostly preferred^5^. Moreover, most current circulating miRNA biomarkers have been discovered from peripheral venous blood due to safety and accessibility. However, while bypassing different tissues or organs, miRNAs in arterial and venous blood might be slightly altered. Our previous work illustrated marginal but nonidentical miRNAs in matched arterial and venous plasma under physiological conditions^6^. Further analysis also found a significant correlation between arterial highly expressed miRNA and specific tissue enriched miRNA expression^6^. In this study, we sought to explore whether deregulated miRNAs could be identified and their differences are amplified under a given disease condition. If it occurs, it would be necessary tore-evaluate the previous markers derived from venous circulation. Therefore, the differential expression of miRNAs between venous and arterial tissues cannot be neglected under the precision medicine background.

Hypertension, a disease that is particularly centered on vessels, was chosen as our disease model. The peripheral arteriole can be affected by long-term force of blood pushing against the artery walls, and its stenosis and/or reduced wall elasticity leads to high blood pressure. Nevertheless, the veins are rarely involved^7^. Spontaneously hypertensive rats (SHRs) which derived from WKY strain are the most widely studied animal model of essential hypertension, and have been extensively used to study cardiovascular diseases (CVDs). Similar to humans, this strain of rats starts hypertensive response at advancing age and the cause of the rising blood pressure remains unknown. Blood pressure of these animals begins to increase at the age of 5 weeks and reaches a hypertensive plateau at approximately 15–16 weeks^8^, when the rats develop characteristics of high blood pressure without other cardiovascular disease (i.e., vascular and cardiac hypertrophy).

In the present study, to explore whether peripheral venous blood miRNAs could represent a bona fide specific miRNA deregulation pattern, we selected hypertension, a disease that is particularly associated with vessels, as the model. Circulating miRNA profiles of arterial and venous plasma were firstly explored in animal models of 16-weeks-old SHR (i.e. SHR group) and Wistar–Kyoto controls (i.e. WKY group). Using the small RNA sequencing technique, changes in circulating miRNAs of WKY-vs-SHR differences and arterial-vs-venous differences were explored. Potential target genes and their regulatory mechanisms were analyzed by MSigDB and TransmiR bioinformatics tools, respectively, to unveil the underlying cause and function of these differential miRNAs. Moreover, circulating arterial and venous miRNA profiles from 5-weeks-old SHR (i.e. JSHR group) were also sequenced and applied as additional validation controls. The differentially expressed miRNAs between SHR and JSHR were measured, and the shared de-regulated miRNAs between SHR-WKY comparison and SHR-JSHR comparison in arteries and veins were analyzed as potential biomarker candidates for hypertension.

## Methods

### Animals

Male SHRs and WKY rats were purchased from Beijing Vital River Laboratory Animal Technology Co., Ltd. Animals were accommodated in standard conditions (temperature 21 ± 2 °C, 12:12 light–dark cycle, lights on at 8:00 am) with free access to food and water. After one week of rest, the systolic, diastolic and mean blood pressures of the rats were measured weekly by a noninvasive tail-cuff method. Briefly, an animal sphygmomanometer (BP98AWU, Softron, Japan) was performed on conscious animals with multiple readings. Data were obtained until 3 stable measurements were obtained in a row, and the average of blood pressure values was calculated. Animal experiments were performed in accordance with the *Guidelines for Animal Experiment* and approved by the Animal Care Committee of the Peking University Third Hospital.

### MiRNA expression profiling of SHR group and WKY group rats by small RNA sequencing

16-Weeks-old SHR (i.e. SHR group) and WKY (i.e. WKY group) animals were anesthetized by pentobarbital sodium, and arterial and venous blood samples were collected simultaneously on one occasion from the abdominal aorta and postcava, respectively. Blood was transferred to EDTA anticoagulation tubes. Next, plasma was obtained by centrifugation, followed by RNA isolation with a miRNeasy Serum/Plasma Kit (Qiagen) and sequencing with an Illumina HiSeq sequencer (service provided by Novogene, Beijing, China). Five biological replicates were performed for small RNA sequencing for each group. After quality control and screening against contaminant and adaptor sequences, the clean reads were aligned to the rat genome (Ensembl Rnor_6.0) by the bowtie tool, and the expression levels of known miRNAs from miRBase (http://www.mirbase.org, release 21) were quantified and normalized in TPM by RSEM^9^. Finally, differentially expressed miRNAs with a p-value < 0.05 and fold change (FC) > 1.5 were obtained by the DEseq2 tool^10^ using the recommended parameters. The differential expression analysis results were also validated by the analysis-of-variance (ANOVA)with the additional consideration of the interaction between genotype (SHR-vs.-WKY) and sample source (Arterial blood-vs.-Venous blood).

### MiRNA expression profiling of the JSHR group rats

Arterial and venous blood samples from 5-weeks-old SHR rats (i.e. JSHR group) were collected and prepared following the same protocol that has been described above. The sequencing was performed with an Illumina HiSeq sequencer (service provided by Shanghai Biotechnology Corporation, Shanghai, China). The alignment, gene expression estimation and differential expression analysis were also performed following similar protocol as described above, but an additional batch effect removal was conducted using the well-established Combat/SVA algorithm^11^ before differential expression analysis.

### Functional enrichment analysis of differentially expressed miRNAs and their Targets

The TAM 2.0 server^12^ was used to analyze the significant functional and disease associations of the differentially expressed miRNAs. We noted that the reference miRNA sets of TAM 2.0 were overwhelmed by cancer-related disease terms. To focus on the associations between artery circulating miRNAs and cardiovascular diseases, we masked the cancer-related terms and the nonstandard terms when executing the analysis task. In addition to regular overrepresentation analysis, TAM 2.0 also allows the comparison of differentially expressed miRNAs with deregulated miRNAs in other diseases. When performing this comparison analysis, the cancer-related terms and the nonstandard terms were also masked.

We also sought to investigate the regulatory interactions between differentially expressed miRNAs and other coding genes. First, the potential target genes of the differentially expressed miRNAs were predicted by PITA^13^, RNAhybrid^14^ and miRanda^15^. Only the consensus targets shared by at least two predictors were retained. Intensively regulated genes that were targeted by more than three differentially expressed miRNAs were selected for further functional enrichment analysis. The functional enrichment analysis was performed by the investigation tool provided by MSigDB^16^ using default parameters and its hallmark gene sets as the reference. The results were visualized as heatmaps with the ‘pheatmap’ package in R. In addition to the downstream target genes, the upstream transcription factors (TFs) that tended to regulate the differentially expressed miRNAs were screened by using the TransmiRv2.0 tool^17^. We used the high confidence TF-miRNA sets (i.e., the ‘set level 2’) as the reference. The interactions between differentially expressed miRNAs and top TFs were visualized by the network visualization tool Cytoscape (http://www.cytoscape.org). For all functional enrichment analyses performed by TAM 2.0 and TransmiR v2.0, the p-values were FDR corrected by the Benjamini–Hochberg method.

### ddPCR validation of differentially expressed miRNAs

For reverse transcription of plasma miRNA, a RevertAid First Strand cDNA Synthesis kit (Thermo, CA) was applied. Detection and quantification of deregulated miRNAs was analyzed by droplet digital PCR (ddPCR) (Bio-Rad Laboratories, Hercules, CA, USA). Briefly, ddPCR reaction volumes were prepared using the QX200 ddPCR system according to the manufacturer’s instructions, and data were analyzed by QuantaSoft software (Bio-Rad Laboratories).

### Statistical analysis for blood pressure and ddPCR assay

Data are expressed as the mean ± SD. Comparisons between groups were evaluated using a two-tailed Student’s t test and p < 0.05 was considered statistically significant.

## Results

### Magnified deregulated miRNAs between arterial blood and venous blood in hypertensive condition

In this study, we first measured circulating miRNA profiles in 16-weeks-old SHR (i.e. SHR group) and their age-matched controls (i.e. WKY group) (Supplementary Fig. S1). Venous and arterial circulating miRNAs from postcava and abdominal aorta were obtained simultaneously on one occasion from these two groups, and then analyzed by the small RNA sequencing pipeline.

**Figure 1:**
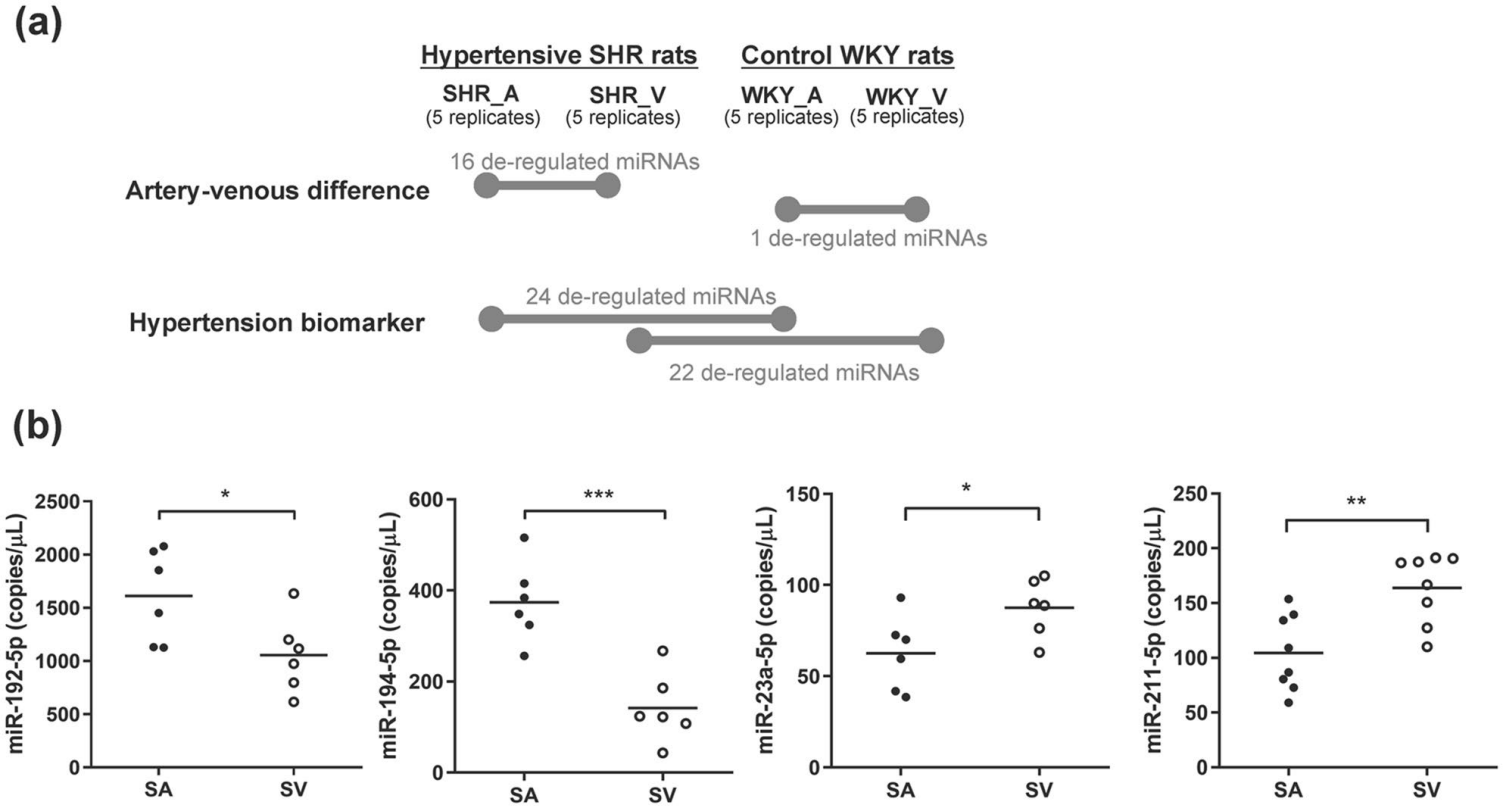
Magnified deregulated miRNAs of artery-vs-vein in hypertension. (**a**) Overview of the differential miRNAs. (**b**) Quantification of rno-mir-192-5p, rno-mir-194-5p, rno-mir-23a-5p and rno-mir-211-5p in arterial and venous plasma of 6 SHRs by droplet digital PCR. SV: venous blood from SHRs; SA: arterial blood from SHRs. *P < 0.05, **P < 0.01, ***P < 0.001 vs. SA.

Only one deregulated miRNA was observed between arterial and venous circulating miRNAs of WKY rats (Fig. 1a, Supplementary Table S1). This miRNA, rno-miR-483-3p, showed higher expression in veins (WKY_A vs. WKY_V). This result is consistent with a previous study showing that miR-483-3p negatively regulates angiogenesis and is upregulated in endothelial progenitor cells of deep vein thrombosis patients^18^. In addition, this miRNA has been reported to be associated with several other cardiovascular diseases of ischemic cardiomyopathy and atrial fibrillation, as recorded in the HMDD v3.0 database^19^.

The numbers of arterial-vs-venous de-regulated circulating miRNAs are enhanced in the SHR hypertensive model, with about half of the deregulated miRNAs confirmed by both the pairwise and the ANOVA statistical tests (Supplementary Table S1). Among these deregulated miRNAs, 6 showed higher expression in arteries, while 10 showed higher expression in veins (Fig. 1a). MiRNAs of rno-miR-15b-5p, rno-miR-122-3p, rno-miR-30a-5p, rno-miR-505-3p, rno-miR-326-3p, rno-let-7d-3p, rno-miR-211-5p, rno-miR-194-5p, rno-miR-192-5p and rno-miR-23a-5p have been reported to be associated with CVDs (accounting for two-thirds of the total numbers), and the first five (rno-miR-15b-5p, rno-miR-122-3p, rno-miR-30a-5p, rno-miR-505-3p, rno-miR-326-3p) are related to hypertension^20–25^. These 5 miRNAs biomarkers are identified from the venous plasma in previous studies. Further mechanistic insights into hypertension-associated pathogenesis indicated that miR-30a stimulates arteriolar branching morphogenesis by regulating endothelial tip cell formation^22^, while miR-505 negatively modulates angiogenic processes by impairing the migration and tube formation of endothelial cells^23^. These previous studies support our small RNA sequencing results that miR-30a is higher in arteries and miR-505 is higher in veins in SHR models. Some other deregulated miRNAs associated with CVD but not hypertension (rno-let-7d-3p, rno-miR-211-5p, rno-miR-194-5p, rno-miR-192-5p and rno-miR-23a-5p). The de-regulation of these miRNAs were further validated by droplet digital PCR (ddPCR) in arterial and venous plasma of SHRs. Arterial highly expressed miRNAs (rno-miR-192-5p, rno-miR-194-5p) and venous highly expressed miRNAs (rno-miR-23a-5p, rno-miR-211-5p) were validated in 6 SHRs, with significant arteries-versus-veins differences (Fig. 1b). The consistent expression patterns of ddPCR and miRNA sequencing support the reliability of the transcriptome data, and clinical potential biomarker of hypertension.

### Functions and disease association of the arterial blood-vs-venous blood deregulated miRNAs in hypertension

**Figure 2:**
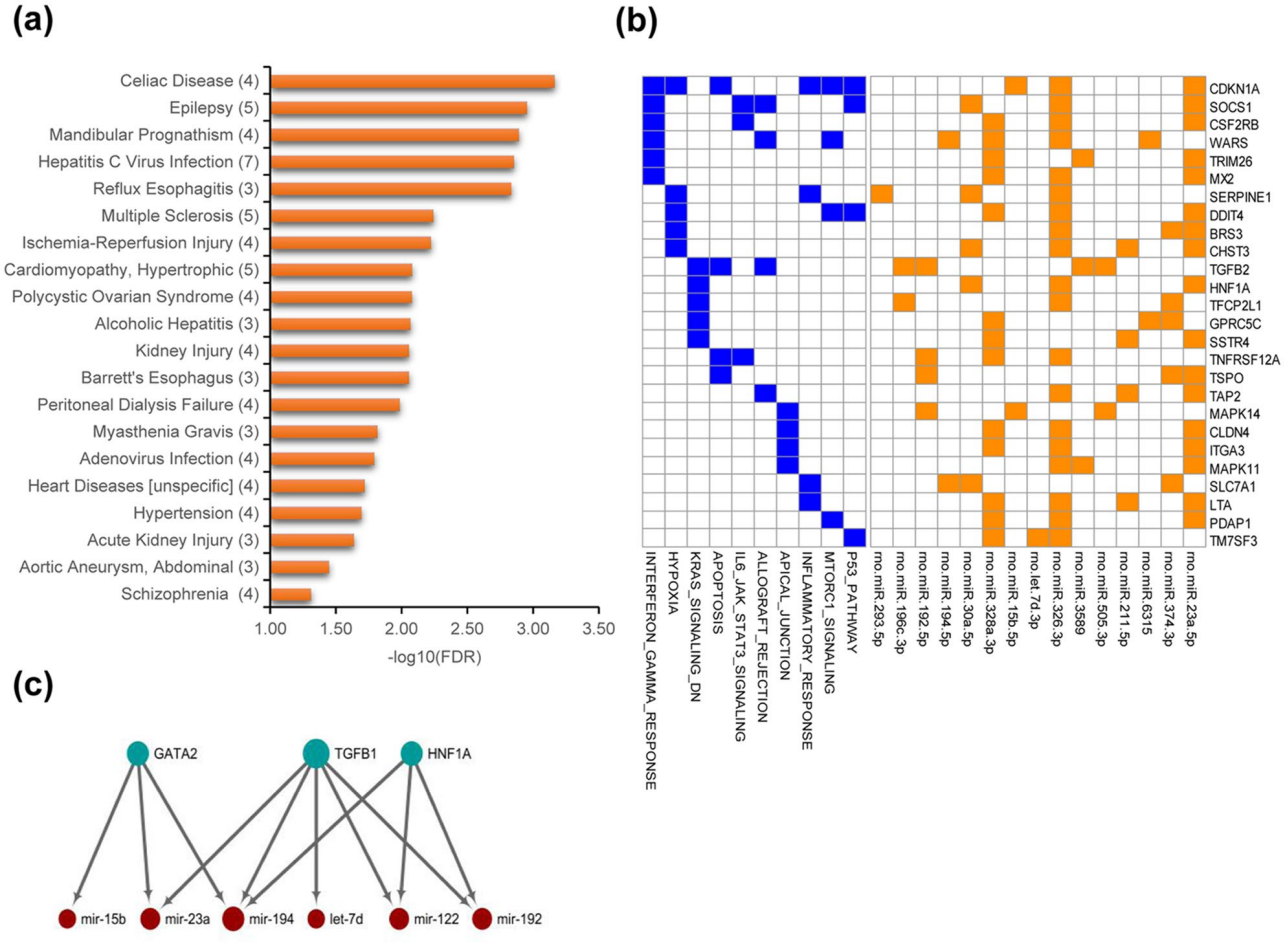
Functions and disease association of these arteries-vs-veins deregulated miRNAs in hypertension. (**a**) Top 20 significant disease associations of the deregulated miRNAs in the arteries-vs-veins of SHR. (**b**) Heatmaps of target genes of hypertensive arteries-vs-veins miRNAs and their enriched pathways. Genes are targeted by at least three differentially expressed miRNAs. The interactions of miRNAs and target genes are indicated by orange boxes, while the presence of the target gene in certain pathways is indicated by blue boxes. The binary heatmap shows all genes targeted by three miRNAs. (**c**) Regulatory network of enriched upstream transcription factors and their regulated miRNAs.

To explore the potential applications of these hypertensive arterial-versus-venous circulating miRNAs, we analyzed the overrepresented disease associations using the TAM 2.0 tool. The top20 significant disease terms are shown in Fig. 2a. It is noteworthy that TAM 2.0 used manually curated miRNA-disease associations from literature as its reference miRNA sets, and thus has intrinsic literature bias (i.e. some diseases like cancers have much more associated miRNAs while some other diseases have only few associated miRNAs), resulting in false positive and negative results. Nevertheless, we noted that hypertension was observed in this disease association graph (FDR = 2.05E−2). In addition, a few cardiovascular diseases like ischemia–reperfusion injury (FDR = 6.16E−3) and hypertrophic cardiomyopathy (FDR = 8.46E−3) are also found in the list.

Then, we explored genes intensively targeted by at least three of these deregulated miRNAs. As intuitively expected, genes involved in the hypoxia response were targeted (HALLMARK_HYPOXIA, FDR = 4.24E−3, Fig. 2b). Moreover, genes associated with angiogenesis (HALLMARK_P53_PATHWAY, HALLMARK_KRAS_SIGNALING_DN, HALLMARK_APOPTOSIS), vascular permeability (HALLMARK_APICAL_JUNCTION), and immune responses (HALLMARK_INTERFERON_GAMMA_RESPONSE, HALLMARK_IL6_JAK_STAT3_SIGNALING, HALLMARK_ALLOGRAFT_REJECTION, HALLMARK_INFLAMMATORY_RESPONSE) were also in the top enriched gene sets (FDR < 0.05) (Fig. 2b).

Finally, we identified plausible causal factors of these deregulated miRNAs. A regulatory network of three regulatory transcription factors (TFs) and 6 miRNAs was constructed (Fig. 2c). We noted two enriched TFs, TGFB1 and HNF1A, as components of the ALK pathway in cardiac myocytes (i.e., BIOCARTA_ALK_PATHWAY in MSigDB gene sets), which indicates a potential functional link between artery-vs-venous miRNA and heart development functions.

### A nonnegligible association of artery deregulated miRNAs and high blood pressure

**Figure 3:**
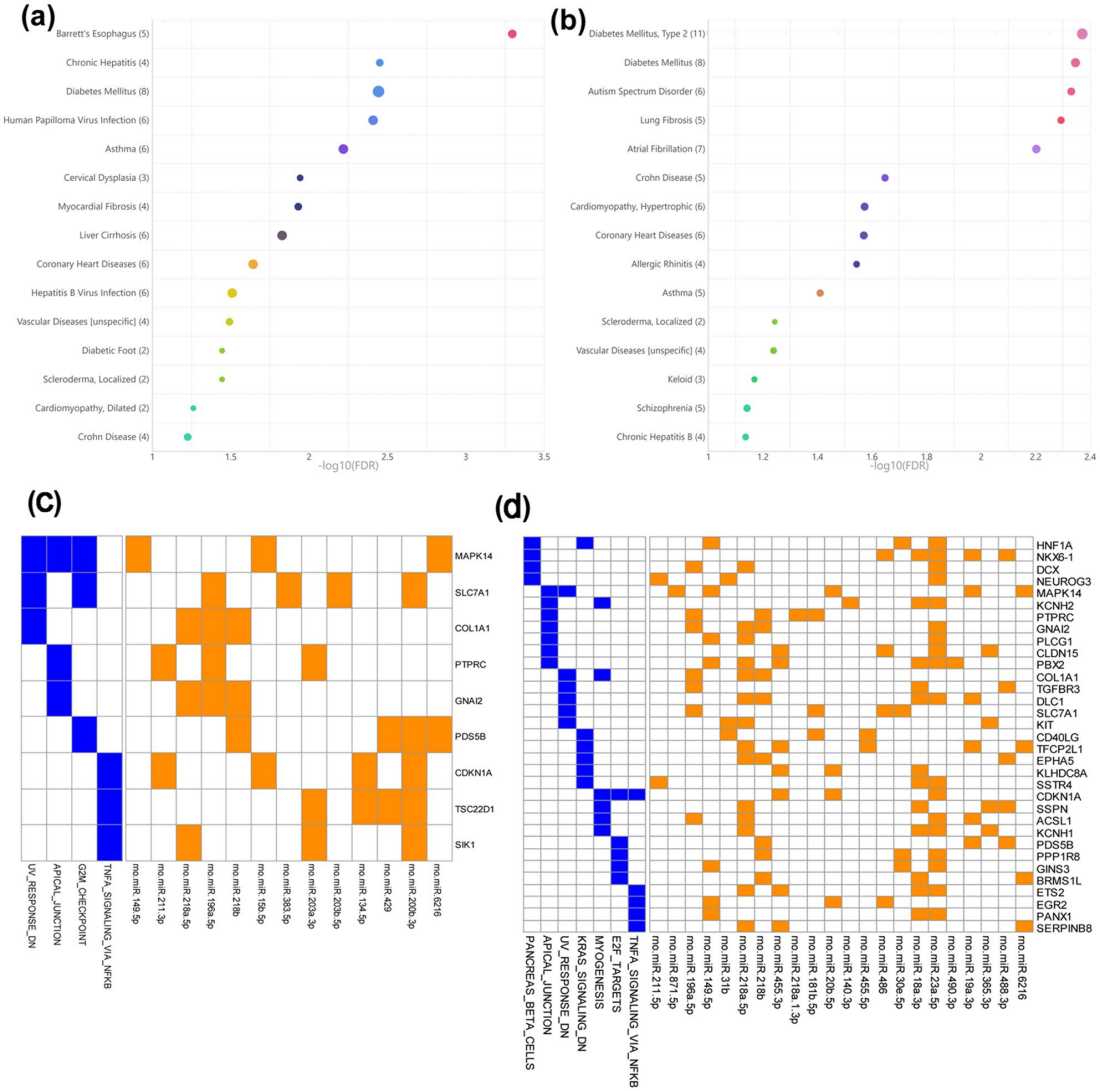
Functions and disease association of the deregulated circulating miRNAs of SHR-vs-WKY comparisons in arterial and venous blood. (**a**,**b**) Disease association of deregulated venous miRNAs (**a**) and deregulated arterial miRNAs (**b**) in SHR-vs.-WKY comparisons. Bubbles are ranked by statistical significance (FDR), and the bubble size is proportional to the number of deregulated miRNAs associated with certain diseases. (**c**,**d**) Deregulated venous circulating miRNAs (**c**) and deregulated arterial circulating miRNAs (**d**) in SHR-vs-WKY comparisons. Interactions of miRNAs and target genes are indicated by orange boxes, and the presence of target genes in pathways is indicated by blue boxes.

While current knowledge of miRNA biomarkers is largely based on circulating venous miRNAs, we first analyzed the differential miRNAs of SHR-versus-WKY in the venous blood. A total of 22 deregulated circulating miRNAs (10 upregulated miRNAs, 12down-regulated miRNAs) were identified and most of these de-regulated miRNAs could be confirmed by either pairwise and ANOVA statistical analyses (Fig. 1a, Supplementary Table S2). Although TAM 2.0 disease association analysis, as above stated, has the problems of false positives and false negatives, analysis of these miRNAs does reveal hypertensive-related cardiovascular diseases like myocardial fibrosis (FDR = 1.14E−2) and coronary heart diseases (FDR = 1.17E−2) (Fig. 3a).

Then, the deregulated arterial circulating miRNAs of SHR-versus-WKY were analyzed. A total of 24 deregulated miRNAs (14 upregulated miRNAs and 10 downregulated miRNAs) were observed and most of these de-regulated miRNAs could be confirmed by two statistical analyses (Fig. 1a, Supplementary Table S2). Among these miRNAs, 5 miRNAs (rno-miR-196a-5p, rno-miR-211-5p, rno-miR-149-5p, rno-miR-218a-5p and rno-miR-218b) overlapped with the deregulated venous circulating miRNAs (accounting for one fifth of the total numbers), with consistent differential expression trends. The disease association of these 24 arterial deregulated miRNAs was similar to that of the 22 venous deregulated miRNAs. For example, the hypertensive-related cardiovascular diseases of coronary heart diseases (FDR = 2.69E−2) (Fig. 3b). However, few other hypertensive-related diseases were present in artery-deregulated miRNAs, such as the cardiovascular disease atrial fibrillation (FDR = 6.28E−3) (Fig. 3b). To further determine the disease associations of these arterial deregulated miRNAs, we compared them with various disease deregulated miRNAs by using the TAM 2.0 tool. Interestingly, artery-deregulated miRNAs showed a positive correlation with viral myocarditis and negative correlations with atrial fibrillation and congenital heart diseases (Supplementary Fig. S2b). Such correlations of deregulated miRNAs with cardiovascular diseases were not observed for venous deregulated miRNAs (Supplementary Fig. S2a). Together, the above analysis indicated a nonnegligible association of artery-deregulated miRNAs and high blood pressure.

**Figure 4:**
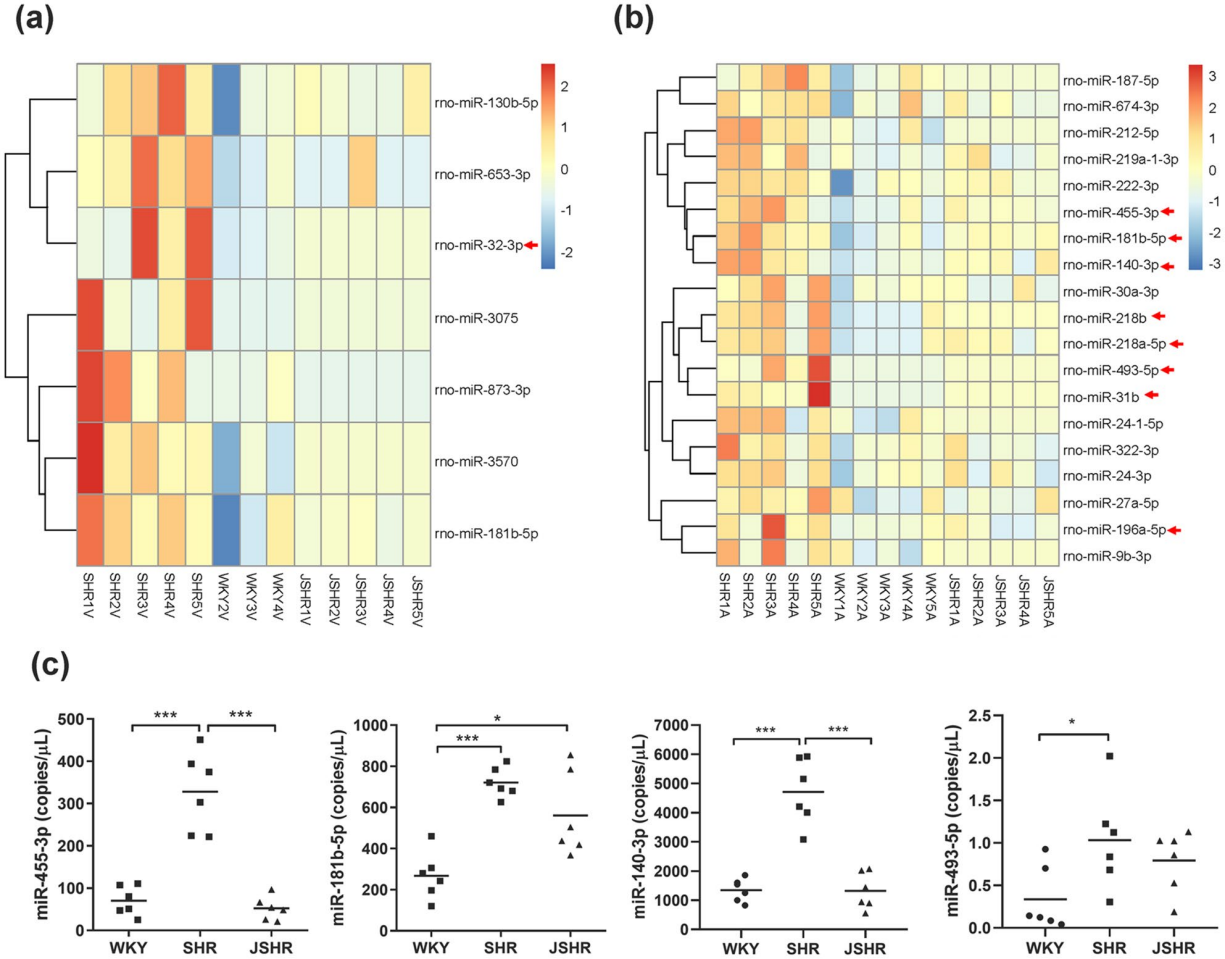
Elevated arterial circulating microRNAs might be potential biomarker for hypertension. (**a**,**b**) Overlaps of deregulated miRNAs from SHR-vs-JSHR comparison and SHR-vs-WKY comparison in venous blood samples (**a**) and arterial blood samples (**b**). The heatmap shows all of the shared miRNAs. Note that batch effect correction was applied to combine the expression profiles of SHR/WKY groups and that of the JSHR group (because JSHR was sequenced in another batch). As the result, the differentially expressed in this analysis should be differed from the previous SHR-vs-WKY comparison. Nevertheless, 9 miRNAs are shared with the previous analysis (8 from arterial blood and 1 from venous blood), and these 9 miRNAs are highlighted by red arrows aside the heatmaps. (**c**) Droplet digital PCR quantification of rno-miR-455-3p, rno-miR-181b-5p rno-miR-140-3p and rno-miR-493-5p in arterial plasma of 6 WKY, 6 SHR and 6 JSHR rats. *P < 0.05, ***P < 0.001.

We further analyzed the target genes and the enriched functions of these arterial circulating miRNAs of SHR-vs-WKY. Despite the limited overlap of differential miRNAs between arteries and veins (SHR_A-vs-WKY_A and SHR_V-vs-WKY_V) (Supplementary Table S2), their intensively regulated targets were noticeably partially shared. Seven out of nine (77.7%) miRNAs (MAPK14, SLC7A1, COL1A1, PTPRC, GNAI2, PDS5B, and CDKN1A) in the SHR_V-vs-WKY_V comparison were observed in the SHR_A-vs-WKY_A comparison, although the arterial circulating miRNAs had more targets (Fig. 3c,d). In line with the shared target genes, enriched pathways were also prominently overlapped between these two comparisons, i.e., genes downregulated in response to ultraviolet radiation (HALLMARK_UV_RESPONSE_DN), genes encoding components of the apical junction complex (HALLMARK_APICAL_JUNCTION) and genes regulated by NF-κB in response to TNF-α (HALLMARK_TNFA_SIGNALING_VIA_NFKB) (FDR < 0.05, Fig. 3c,d). However, we observed three more enriched functions, namely, cell cycle and angiogenesis (HALLMARK_E2F_TARGETS, HALLMARK_MYOGENESIS, HALLMARK_KRAS_SIGNALING_DN, FDR < 0.05, Fig. 4b) associated with arterial circulating miRNA target genes, which indicated a greater association of arterial miRNAs and hypertension.

### Elevated artery circulating microRNAs might be potential biomarker for hypertension

Blood pressure of SHRs begins to increase at the age of 5 weeks^8^, so, these young non-hypertensive animals (5-weeks-old SHR, i.e. JSHR group) could be an excellent second control for SHR group. Systolic-BP and diastolic-BP of JSHRs are similar with WKYs, but are remarkable lowered than SHRs (Supplementary Fig. S1). Arterial and venous circulating miRNAs from abdominal aorta and postcava were obtained simultaneously on one occasion from these animals, sequenced and analyzed. Because the JSHR group were sequenced in another batch, batch effect correction algorithm was applied before differential expression analysis, and the overlaps between deregulated miRNAs observed in the SHR-vs-JSHR comparison and those observed in the SHR-vs-WKY comparison are identified in venous (Fig. 4a) and arterial blood samples (Fig. 4b).

A total of 19 overlapped arterial deregulated miRNAs were identified. Because these de-regulated miRNAs are observed in both SHR-vs-JSHR and SHR-vs-WKY comparison, they are more likely to be associated with high blood pressure. Among these miRNAs, 8 miRNAs (rno-miR-455-3p, rno-miR-181b-5p, rno-miR-140-3p, rno-miR-218b, rno-miR-218a-5p, rno-miR-493-5p, rno-miR-31b, rno-miR-196a-5p) are shared with the previous SHR_A-vs-WKY_A comparison (the deregulated miRNAs in SHR_A-vs-WKY_A comparison has been listed in Supplementary Table S2), implying higher confidence for their associations with hypertension. By contrast, only 7 overlapped deregulated miRNAs which are likely to be associated with high blood pressure, are identified. Moreover, only one of these miRNAs (rno-miR-32-3p) is shared with in the previous SHR_V-vs-WKY_V comparison (the deregulated miRNAs in SHR_V-vs-WKY_V comparison has also been listed in Supplementary Table S2). In all, among the 9 consistent differentially expressed miRNAs, 8 miRNAs are from arterial blood and 1 miRNA is from venous blood), suggesting more extensive associations of the arterial deregulated miRNAs with hypertension than the venous deregulated miRNAs.

Among the 8 shared high blood pressure related arterial deregulated miRNAs, rno-miR-31b has been reported to be associated with hypertension^26,27^ Other 7 miRNAs have not been validated with respect to their associations with hypertension. Because 3 miRNAs (rno-miR-218b, rno-miR-218a-5p andrno-miR-196a-5p) are not arterial specific de-regulated miRNAs (i.e. also presented in SHR_V-vs-WKY_V comparison, Supplementary Table S2), we here focused on the rest 4 miRNAs (rno-miR-455-3p, rno-miR-181b-5p, rno-miR-140-3p and rno-miR-493-5p) to validate the associations of arterial circulating miRNAs with hypertension. The de-regulation of these four miRNAs in arterial blood during hypertension was confirmed by ddPCR assay. In consistent with the small RNA sequencing analysis results, all these four miRNAs displayed high levels in the arterial plasma of SHRs (Fig. 4c). Moreover, rno-miR-455-3p and rno-miR-140-3p showed significantly reduced levels in JSHRs when compared to SHRs. Thus, the elevated arterial circulating miR-455-3p and miR-140-3p might be potential prognostic markers for hypertension.

## Discussion

Circulating miRNAs have been extensively studied as biomarkers due to their rapid release dynamics and stable existence. While most current circulating miRNA biomarkers have been discovered in venous blood samples, whether these markers are precisely correlated with a certain disease and could represent the actual pathophysiological changes under a given condition needs to be further investigated. Our previous work identified marginal but nonidentical miRNA expression patterns between arteries and veins under physiological conditions^6^. This study took a step further to investigate changes in arterial and venous circulating miRNAs under disease conditions.

First, we compared differential miRNA expression between arterial and venous plasma in WKY rats and SHRs using small RNA sequencing technology. Magnified deregulated circulating miRNAs were identified while blood pressure elevation (16 in SHRs vs. 1 in WKYs). The underlying mechanism of essential hypertension is complex, which associated with the complex molecular network of vascular metabolism, endothelial dysfunction, inflammation, and the renin–angiotensin-aldosterone system (RAAS), etc.^28–30^. Disease association of these 16 differentially expressed miRNAs revealed the involvement of conditions related to hypertension like cardiovascular diseases.

Hypoxia responsive genes were targeted while analyzing the functions of these 16 deregulated miRNAs (Fig. 2b). This can be intuitively understood, because arteries carry oxygenated blood while veins not. It is well known that elevated oxygen levels may induce vascular wall remodeling^31^. Besides, our study also identified hallmarks of target genes of angiogenesis, vascular permeability and inflammation (HALLMARK_P53_PATHWAY, HALLMARK_APOPTOSIS, and MALLMARK_APICAL _JUNCTION), which have been reported to be associated with blood vessels and oxygen levels. The severity and duration of hypoxia are known to modulate the p53 pathway in a manner dependent or independent of the main transcription factor of HIF-1 (hypoxia-inducible factor-1)^32^; hyperbaric oxygen induces apoptosis by activating the p38 MAP-kinase pathway, initiating the extrinsic death receptor pathway or the mitochondrial intrinsic pathway^33–35^. Besides, studies also reveal that ROS can alter vascular permeability both in vitro and in vivo by junctional protein phosphorylation regulation or actin cytoskeleton organization^7^. Together, the above evidence supports the hypertensive function of deregulated miRNAs.

Similar to protein-coding genes, miRNAs transcription is regulated by transcriptional factors (TFs) at the transcriptional level as well^36^. Due to the limited knowledge of TF-miRNA regulations, only three TFs (GATA2, TGFB1, and HNF1A) were identified from these 16 deregulated miRNAs (Fig. 2c). However, these 3 TFs are key events in cardiovascular diseases and may provide clues for causal factors of deregulated miRNA expression in hypertension. Transforming growth factor *beta* (TGFβ), which is essential for proper cardiovascular development, plays critical roles in homeostatic, repair and stress response processes^37^. HNF‐1a could bind to genes whose products are involved in cardiovascular diseases^38^. GATA2 has been reported to be abundantly expressed in vascular endothelial cells (ECs) and has been identified as a key transcription factor that regulates classical markers of EC activation^39^. Of note, the functions of transcriptional factors are more specific and intensive for downstream encoding proteins than miRNAs. Therefore, more investigations that could deepen our understanding of the influence of blood pressure under different conditions from a transcriptional point of view are expected.

A further step was taken to explore changes of arterial and venous circulating miRNAs under hypertension conditions (WKY-vs.-SHR). Though minor overlapped circulating miRNAs (accounting for one fifth of the total numbers, Supplementary Table S2), disease association analysis revealed the similarity between arterial and venous de-regulated miRNAs like cardiovascular diseases and immune diseases (Fig. 3a,b, Supplementary Fig. S2). Hypertension is quantitatively the most important modifiable risk factor for premature cardiovascular disease^40^, and the immune system plays a prominent role in the initiation and maintenance of hypertension^29^. But beyond that, certain disease terms were uniquely represented in arterial differentiated miRNAs. Moreover, functional analysis of these differential miRNAs also identified more targeted genes specifically correlated with arterial deregulated miRNAs (Fig. 3c,d). The gene of BRMS1L plays a role in regulating Wnt signaling^41^, the gene of HNF1A is a component of angiogenesis regulators^42^, and the homeobox β-cell transcription factor of NKX6-1 could induce β-cell proliferation^43^. These target genes imply the potential significance of arterial circulating miRNAs and a greater association of arterial deregulated miRNAs with cardiovascular diseases.

While current knowledge of miRNAs biomarkers is largely based on circulating venous miRNAs, our study implied a rationality of arterial circulating miRNAs and the possibility of identifying potential hypertension prognostic biomarkers in arterial blood. After intensive selection, 4 miRNAs, includingrno-miR-455-3p, rno-miR-181b-5p, rno-miR-140-3p andrno-miR-493-5p are prioritized. The de-regulation of these miRNAs have been supported by (a) SHR_A-vs-WKY_A comparison (Supplementary Table S2); (b) SHR-vs-JSHR and SHR-vs-WKY comparisons after batch effect correction (Fig. 4b) and (c) ddPCR validation assay. Therefore, these 4 miRNAs could serve as miRNA biomarker candidates for hypertension prognostics. Interestingly, although none of these 4 miRNAs has been reported to be associated with hypertension in literature, rno-miR-455-3p, rno-miR-181b-5p, rno-miR-140-3p were reported to be CVD-related. miR-181b-5p was relevant with different kinds of CVDs, such as atherosclerosis^44^, abdominal aortic aneurysm^45^, aortic valve disease^46^, hypertrophic cardiomyopathy^47^. miR-455-3p could be a therapeutic success biomarker of the aneurysmal sac exclusion after endovascular treatment^48^, and miR-140-3p is a potential biomarker for CVD patients receiving dexmedetomidine^49^. Together, the elevated arterial circulating miR-455-3p and miR-140-3p might be potential prognostic markers for hypertension. This interesting results suggested the possibility of identifying potential prognostic biomarkers for hypertension among arterial circulating miRNAs, in addition to the conventional venous circulating miRNAs.

In previous studies, Hermann et al. explored transcriptomic profiling of cell-free and vesicular microRNAs from matched arterial and venous sera of patients scheduled for cardiac surgery^50^. Hermann’s study is quite helpful for providing a further understanding of severe heart diseases. Since the insertion of arterial lines is more convenient for critically ill patient monitoring, the authors chose the radial artery and internal jugular vein for sample collection. However, there are some differences between their study and ours. First, they focused more on miRNAs in extracellular vesicles instead of those carried by proteins. Second, the miRNA profiles may also be different between plasma and serum. Serum carries a great amount of miRNAs derived from platelets.

Bypassing different organs or tissues, it is conceivable that miRNAs might be slightly altered between veins and arteries. Of note, these changes could not be neglected because the slight difference might be magnified in certain physiological and pathological conditions, such as hypertension, aging and cancer. Therefore, our study indicates that it is necessary to re-evaluate the efficacy of certain miRNAs originating from veins, which have been identified in a previous study as potential biomarkers in cardiovascular diseases. Moreover, this phenomenon might exist even in wider spectrum diseases in addition to hypertension. These findings may promote the progression toward an era of more precise and accurate liquid biopsies.

## Supplementary Information

Supplementary Information.
