# Supplementary material for: Transcriptome analysis of arterial and venous circulating miRNAs during hypertension
Source: Sci Rep. 2021 Feb 10;11:3469. doi: 10.1038/s41598-021-82979-7 (PMC7875986; doi:10.1038/s41598-021-82979-7)
Supplement: Supplementary file 1 — Supplementary Information. [file 41598_2021_82979_MOESM1_ESM.docx]

**Transcriptome analysis of arterial and venous circulating miRNAs during hypertension**

Ling Jin^1,#^, Min Li^2,#^, Hao Wang^1^, Zhongnan Yin^1^, Li Chen^1^, Yang Zhou^2^, Yongzheng Han^2^, QinghuaCui^3^, Yuan Zhou^3,*^, Lixiang Xue^1,*^

^1^Center of Basic Medical Research, Peking University Third Hospital, Beijing 100191, China

^2^Department of Anesthesiology, Peking University Third Hospital, Beijing 100191, China

^3^Department of Biomedical Informatics, School of Basic Medical Sciences, Peking University, Beijing 100191, China

^#^These authors contribute equally

* To whom correspondence should be addressed: LixiangXue (E-mail: lixiangxue@hsc.pku.edu.cn). Correspondence may also be addressed to Yuan Zhou (E-mail: zhouyuanbioinfo@hsc.pku.edu.cn)

**Supplementary Materials**


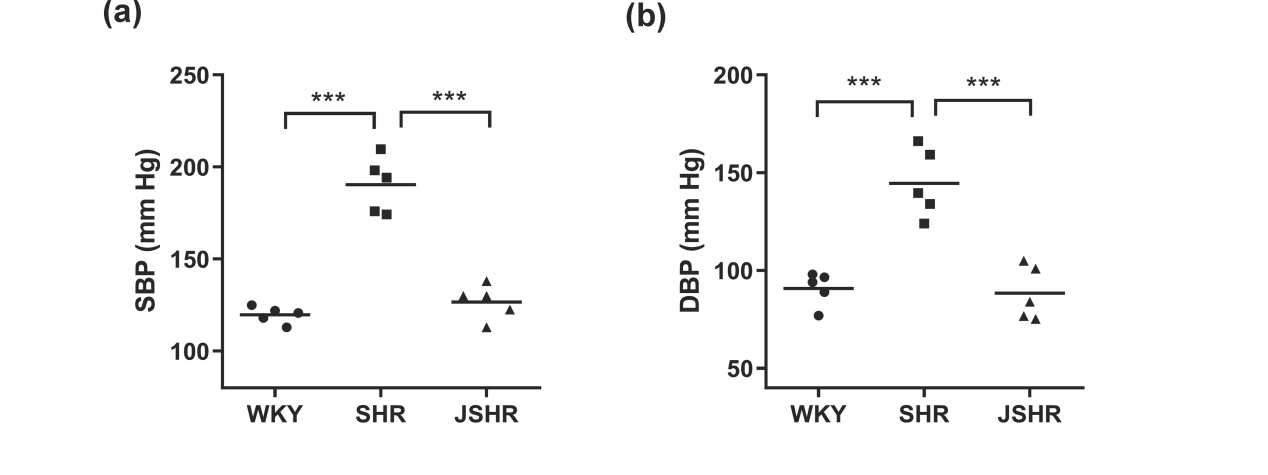


**Supplementary Figure S1. Blood pressure of 5-week-old SHR (JSHR), 16-week-old SHR (SHR) and WKY (WKY) for small RNA sequencing.** (A) systolic blood pressure (SBP) comparison between three groups; (B) diastolic blood pressure (DBP) comparison between three groups.^***^P<0.001 vs. 16w SHR.


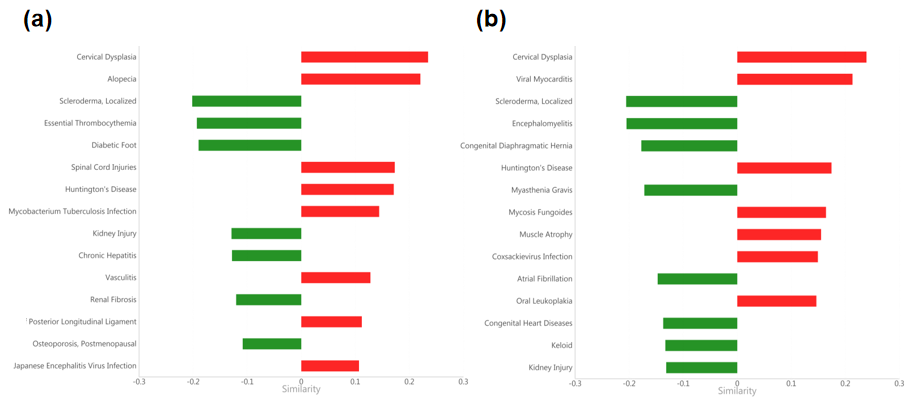


**Supplementary Figure S2.Top 15 disease correlations of de-regulated circulating miRNAs of SHR-vs.-WKY in artery and vein.** Disease correlations of (a) de-regulated venous miRNAs and (b) de-regulated arterial miRNAs in SHR-vs.-WKY comparison. Red bar: positive relationship, where the direction of de-regulation is consistent between the analyzed case and the corresponding disease condition(i.e. de-regulated arterial or venous miRNAs in hypertension); Green bar: negative relationship, where the direction of de-regulation is opposite between the analyzed case.Note that TAM2.0 correlation analysis could only plot the top 15 correlated diseases.

**Supplementary Table S1. List of Arterial blood-vs.-Venous blood de-regulated circulating miRNAs from miRNA sequencing**

| **Arterial blood-vs.-Venous blood in WKY** | | | |  | **Arterial blood-vs.-Venous blood inSHR** | | | |
| --- | --- | --- | --- | --- | --- | --- | --- | --- |
| **microRNA** | **Fold change*** | **p-value (pairwise)*** | **p-value (ANOVA)*** |  | **microRNA** | **Fold change*** | **p-value (pairwise)*** | **p-value (ANOVA)*** |
| rno-miR-483-3p | 0.51 | 0.035 | 0.026 |  | rno-miR-293-5p | 2.47 | 0.007 | 0.038 |
|  |  |  |  |  | rno-miR-196c-3p | 1.88 | 0.049 | n.s. |
|  |  |  |  |  | rno-miR-122-3p ^&^ | 1.77 | 0.026 | n.s. |
|  |  |  |  |  | rno-miR-192-5p | 1.72 | 0.020 | n.s. |
|  |  |  |  |  | rno-miR-194-5p | 1.60 | 0.047 | n.s. |
|  |  |  |  |  | rno-miR-30a-5p ^&^ | 1.38 | 0.033 | n.s. |
|  |  |  |  |  | rno-miR-328a-3p ^&^ | 0.60 | 0.037 | 0.044 |
|  |  |  |  |  | rno-miR-15b-5p | 0.59 | 0.049 | n.s. |
|  |  |  |  |  | rno-let-7d-3p | 0.59 | 0.025 | 0.034 |
|  |  |  |  |  | rno-miR-326-3p | 0.56 | 0.042 | n.s. |
|  |  |  |  |  | rno-miR-3589 | 0.55 | 0.030 | 0.025 |
|  |  |  |  |  | rno-miR-505-3p ^&^ | 0.55 | 0.030 | 0.025 |
|  |  |  |  |  | rno-miR-211-5p | 0.51 | 0.044 | n.s. |
|  |  |  |  |  | rno-miR-6315 | 0.49 | 0.042 | n.s. |
|  |  |  |  |  | rno-miR-374-3p | 0.48 | 0.029 | 0.033 |
|  |  |  |  |  | rno-miR-23a-5p | 0.47 | 0.027 | 0.024 |

* The fold change and p-value for pairwise differential expression analysis were calculated by the DEseq2 tool.The analysis of variance (ANOVA) analysis comparing different sample source (Arterial blood-vs.-Venous blood) with the additional consideration of the interaction between genotype and sample source was also performed by the DESeq2 tool.n.s., not significant.

^&^ miRs that have been described in other hypertension studies.

**Supplementary Table S2. List of SHR-vs.-WKY de-regulated circulating miRNAs from miRNA sequencing**

| **SHR-vs.-WKY in Arterial blood** | | | |  | **SHR-vs.-WKY inVenous blood** | | | |
| --- | --- | --- | --- | --- | --- | --- | --- | --- |
| **microRNA** | **Fold change*** | **p-value (pairwise)*** | **p-value (ANOVA)*** |  | **microRNA** | **Fold change*** | **p-value (pairwise)*** | **p-value (ANOVA)*** |
| rno-miR-211-5p^#^ | 2.92 | 0.002 | 0.004 |  | rno-miR-211-5p^#^ | 3.90 | 0.001 | 0.001 |
| rno-miR-871-5p | 2.91 | 0.002 | 0.005 |  | rno-miR-32-3p | 2.48 | 0.023 | 0.025 |
| rno-miR-196a-5p^#^ | 2.44 | 0.010 | 0.004 |  | rno-miR-149-5p^#^ | 2.43 | 0.008 | 0.005 |
| rno-miR-493-5p | 2.34 | 0.012 | 0.012 |  | rno-miR-211-3p | 2.39 | 0.025 | 0.013 |
| rno-miR-149-5p^#^ | 2.19 | 0.006 | 0.007 |  | rno-miR-218a-5p^#^ | 2.24 | 0.018 | 0.031 |
| rno-miR-31b ^&^ | 2.18 | 0.021 | n.s. |  | rno-miR-196a-5p^#^ | 2.22 | 0.026 | n.s. |
| rno-miR-218a-5p^#^ | 2.14 | 0.020 | 0.012 |  | rno-miR-218b^#^ | 2.22 | 0.020 | 0.032 |
| rno-miR-218b^#^ | 2.12 | 0.021 | 0.014 |  | rno-miR-708-3p | 2.16 | 0.022 | n.s. |
| rno-miR-455-3p | 2.08 | 0.027 | 0.034 |  | rno-miR-15b-5p | 2.16 | 0.030 | 0.035 |
| rno-miR-218a-1-3p | 1.98 | 0.036 | n.s. |  | rno-miR-383-5p | 2.06 | 0.048 | 0.021 |
| rno-miR-181b-5p | 1.97 | 0.020 | 0.016 |  | rno-miR-122-3p ^&^ | 1.79 | 0.049 | n.s. |
| rno-miR-20b-5p | 1.95 | 0.040 | 0.046 |  | rno-miR-203a-3p | 0.56 | 0.048 | n.s. |
| rno-miR-140-3p | 1.92 | 0.003 | 0.008 |  | rno-miR-203b-5p | 0.51 | 0.047 | n.s. |
| rno-miR-455-5p | 1.79 | 0.040 | n.s. |  | rno-miR-143-3p ^&^ | 0.53 | 0.049 | n.s. |
| rno-miR-10a-5p | 0.58 | 0.050 | n.s. |  | rno-miR-450a-5p | 0.50 | 0.042 | n.s. |
| rno-miR-486 | 0.57 | 0.044 | 0.046 |  | rno-miR-134-5p | 0.49 | 0.031 | 0.020 |
| rno-miR-30e-5p | 0.55 | 0.027 | 0.030 |  | rno-miR-450b-5p | 0.49 | 0.034 | 0.042 |
| rno-miR-18a-3p | 0.50 | 0.029 | 0.013 |  | rno-miR-429 | 0.47 | 0.022 | 0.046 |
| rno-miR-23a-5p | 0.49 | 0.046 | 0.028 |  | rno-miR-200b-3p | 0.46 | 0.020 | 0.050 |
| rno-miR-490-3p | 0.47 | 0.027 | 0.050 |  | rno-miR-143-5p ^&^ | 0.44 | 0.027 | 0.043 |
| rno-miR-19a-3p ^&^ | 0.46 | 0.014 | 0.036 |  | rno-miR-145-3p ^&^ | 0.40 | 0.011 | 0.009 |
| rno-miR-365-3p | 0.45 | 0.008 | 0.008 |  | rno-miR-6216 | 0.35 | 0.008 | 0.002 |
| rno-miR-488-3p | 0.42 | 0.009 | 0.016 |  |  |  |  |  |
| rno-miR-6216 | 0.29 | 0.001 | 0.002 |  |  |  |  |  |

* The fold change and p-value for pairwise differential expression analysis were calculated by the DEseq2 tool. The analysis of variance (ANOVA) analysis comparing different sample source (Arterial blood-vs.-Venous blood) with the additional consideration of the interaction between genotype and sample source was also performed by the DESeq2 tool. n.s., not significant.

^#^ miRNAs de-regulated in both of SHR_A-vs-WKY_A and SHR_V-vs-WKY_V comparisons.

^&^ miRs that have been described in other hypertension studies.
